# On the functions of the h subunit of eukaryotic initiation factor 3 in late stages of translation initiation

**DOI:** 10.1186/gb-2007-8-4-r60

**Published:** 2007-04-17

**Authors:** Byung-Hoon Kim, Xue Cai, Justin N Vaughn, Albrecht G von Arnim

**Affiliations:** 1 Department of Biochemistry, Cellular and Molecular Biology, The University of Tennessee, Knoxville, TN 37996-0840, USA; 2 Department of Cell Biology, The University of Oklahoma Health Sciences Center, Stanton L Young Blvd, Oklahoma City, OK 73104, USA

## Abstract

**Background:**

The eukaryotic translation initiation factor 3 (eIF3) has multiple roles during the initiation of translation of cytoplasmic mRNAs. How individual subunits of eIF3 contribute to the translation of specific mRNAs remains poorly understood, however. This is true in particular for those subunits that are not conserved in budding yeast, such as eIF3h.

**Results:**

Working with stable reporter transgenes in *Arabidopsis thaliana *mutants, it was demonstrated that the h subunit of eIF3 contributes to the efficient translation initiation of mRNAs harboring upstream open reading frames (uORFs) in their 5' leader sequence. uORFs, which can function as devices for translational regulation, are present in over 30% of *Arabidopsis *mRNAs, and are enriched among mRNAs for transcriptional regulators and protein modifying enzymes. Microarray comparisons of polysome loading in wild-type and *eif3h *mutant seedlings revealed that eIF3h generally helps to maintain efficient polysome loading of mRNAs harboring multiple uORFs. In addition, however, eIF3h also boosted the polysome loading of mRNAs with long leaders or coding sequences. Moreover, the relative polysome loading of certain functional groups of mRNAs, including ribosomal proteins, was actually increased in the *eif3h *mutant, suggesting that regulons of translational control can be revealed by mutations in generic translation initiation factors.

**Conclusion:**

The intact eIF3h protein contributes to efficient translation initiation on 5' leader sequences harboring multiple uORFs, although mRNA features independent of uORFs are also implicated.

## Abstract 2

Reporter transgene assays and comparative polysome-microarray analysis reveal that the intact h subunit of Arabidopsis eIF3 contributes to efficient translation initiation on mRNA leader sequences harbouring multiple uORFs.

## Background

The eukaryotic translation initiation factor 3 (eIF3) consists of up to 13 recognized subunits and coordinates many of the events leading to start codon recognition by the small ribosomal subunit during the canonical 5' cap-dependent scanning mode of translation initiation [1-5]. The budding yeast eIF3 is simpler, since only five universally conserved subunits form a so-called core complex [6]. Plant eIF3 complexes were purified with 12 distinct subunits [7] and, although recognizable in the *Arabidopsis *genome sequence, homologs of eIF3j are not tightly associated with plant eIF3. The classic functions ascribed to eIF3 are threefold and include: facilitating the charging of the 40S ribosomal subunit with the ternary complex (eIF2, Met-tRNA^Met^, GTP); bridging between the 40S ribosomal subunit and the eIF4G subunit of the cap-binding complex, eIF4F; and inhibiting the association of 40S and 60S ribosomal subunits [3,8]. These events occur prior to establishment of the 48S complex between the 40S subunit and the mRNA and would, therefore, apply equally to every mRNA. Yet, eIF3 remains attached to the 40S ribosome during scanning and is dislodged only during subunit joining [2,3], which opens up the possibility that eIF3 or its subunits affect initiation in an mRNA specific fashion. There is a conceptual precedent for this possibility, as eIF3 interacts with certain internal ribosome entry sites (for example, [9]).

Roles of eIF3 downstream of 48S complex formation are of great interest because they may reveal mRNA selective functions of eIF3, yet these are only beginning to be understood. For example, certain mutations in budding yeast eIF3 subunits c and b cause defects in scanning and AUG start codon recognition [10-12]. In fission yeast, where the eIF3 subunit composition generally conforms to that in multicellular eukaryotes, it was possible to reveal two subtypes of eIF3 that differ with respect to the presence of the eIF3e and eIF3m subunits, and associate with different subsets of mRNAs [13]. The mammalian eIF3e subunit is bound by p56 protein, a cellular component of the antiviral defense, which can shift the balance between host and viral mRNA translation [14]. At the biochemical level, the eIF3 protein complex appears to serve as a docking site for at least two protein kinases that control the translation initiation machinery, the target-of-rapamycin (TOR) kinase, and ribosomal protein S6 kinase [15,16]. eIF3 and its subunits are also thought to contribute to the non-canonical translation initiation of plant viral mRNAs, by binding to a transactivator of ribosome shunting/re-initiation in cauliflower mosaic virus [17,18]. Finally, our lab has documented that carboxy-terminal truncations of the *Arabidopsis *eIF3h protein compromise efficient translation of a subset of mRNAs that harbor upstream open reading frames (uORFs) in their 5' leader sequence, effects that may underlie the pleiotropic phenotypic spectrum of the *eif3h *mutant plant [19].

Among the diversity of mRNA sequence determinants that poise mRNAs for translational control are uORFs, coding sequences of generally fewer than 50 codons that reside either singly or in small clusters in the 5' leader sequence. uORFs often inhibit translation initiation overall [20-23], and play critical roles in signal-dependent regulation of translation (reviewed in [24,25]). In plants, the polyamine-repressible translation of S-adenosyl-methionine decarboxylase is mediated by a pair of short, amino acid sequence-dependent uORFs [26], whereas translational repression by sucrose is accomplished by a conserved uORF found in the leader of several basic leucine zipper transcription factors [27,28].

In pursuit of our goal to identify functions for individual eIF3 subunits in translation initiation, mutant analysis previously suggested that eIF3h contributes selectively to the translation initiation on specific 5' leader sequences [19]. Two eIF3h-dependent mRNAs contained multiple uORFs, whereas several eIF3h-independent mRNAs contained no uORF or only one uORF. However, the number of genes analyzed did not allow a generalization, and the conclusion was based primarily on a transient reporter gene expression assay. Here we have tested the specific hypothesis that eIF3h generally functions in permitting efficient initiation on 5' leaders harboring multiple uORFs. We now present two additional lines of evidence in its favor, one based on translational reporter genes that are stably integrated into the plant genome of *eif3h *mutant plants, and a second based on transcriptome-wide analysis of the mRNA translation state using polysome microarrays.

## Results

### Transgenic analysis of translational efficiency

**Figure 1:**
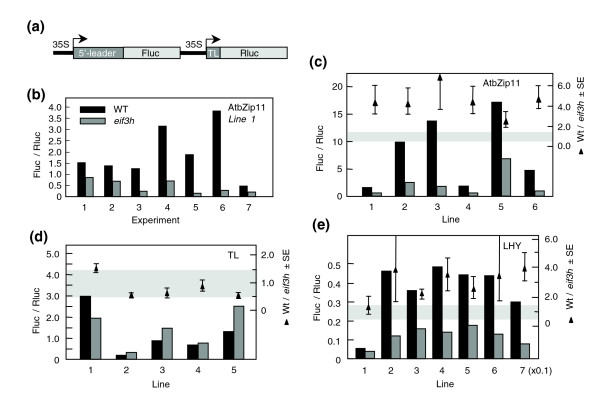
eIF3h controls the translational efficiency of the AtbZip11 leader in stable, transgenic, reporter gene expression cassettes. **(a) **Schematic of the reporter gene T-DNA structure. The efficiency of translation initiation on a given 5' leader sequence is measured by comparing the activity of the associated firefly (Fluc) reporter gene with the activity of the *Renilla *luciferase (Rluc) reference gene, which is expressed under the control of the cauliflower mosaic virus 35S promoter (35S) and the generic 5' leader sequence from tobacco etch virus (TL). **(b) **Translational efficiency of the AtbZip11 (ATB2) leader in wild-type (WT) and *eif3h *mutant seedlings. Seedlings were germinated for nine days on solid agar medium in the light. The figure shows raw Fluc/Rluc activity ratios from seven individual experiments conducted with one transgenic line. The data are representative of other raw data that underlie Figure 1c-e and Figure 2. **(c) **Translational efficiency of the AtbZip11 leader in wild-type (WT) and *eif3h *mutant seedlings. All six independent transgenic lines examined are shown. The bars indicate Fluc/Rluc ratios (left y-axis), while the triangles show the ratio of translational efficiency between wild-type (Wt) and mutant plants (right y-axis). The Wt/*eif3h *bracket between 0.5 and 1.5 is highlighted in gray to facilitate comparison between panels. SE, standard error. **(d) **Translational efficiency of the tobacco etch virus leader (TL) in wild-type (Wt) and *eif3h *mutant seedlings. Data from five lines are displayed as for (c). **(e) **Translational efficiency of the leader of the *Arabidopsis *LHY (At1g01060) gene. Fluc/Rluc bars for line 7 are displayed at 10% of the original values.

To examine how eIF3h contributes to the translation initiation on different 5' leader sequences, reporter gene expression cassettes were introduced as stable transgenes into *Arabidopsis eif3h-1 *mutant and wild-type seedlings. The *eif3h-1 *mutant allele harbors a T-DNA insertion that gives rise to a carboxy-terminally truncated protein [19]. In these transgenes, firefly luciferase (Fluc) reports on the expression of the 5' leader to be tested while *Renilla *luciferase (Rluc), driven by a second copy of the 35S promoter and a generic leader sequence from tobacco etch virus serves as a reference (Figure 1a). The Fluc expression under the control of the 5' leader of AtbZip11 (formerly *ATB2*) was inhibited in the *eif3h *mutant compared to wild-type seedlings, as indicated by the about four-fold elevated Fluc/Rluc activity ratios in the wild type compared to the *eif3h *mutant (Figure 1b,c). The effect of the *eif3h *mutation was consistent (Student's paired *t*-test, *p *< 0.02) in each of the six lines examined (Figure 1c), even though these lines are expected to differ in their luciferase expression level, T-DNA dosage, and the extent of spontaneous gene silencing. Consistent with transient assays reported earlier [19], the data from this new transgenic assay now extend the effect of eIF3h over the entire aggregate of cells in seedling shoots in which the 35S promoter is active, not just the predominantly epidermal cells hit by particle bombardment. The AtbZip11 leader consistently drove higher translation in the wild type than in the mutant.

**Figure 2:**
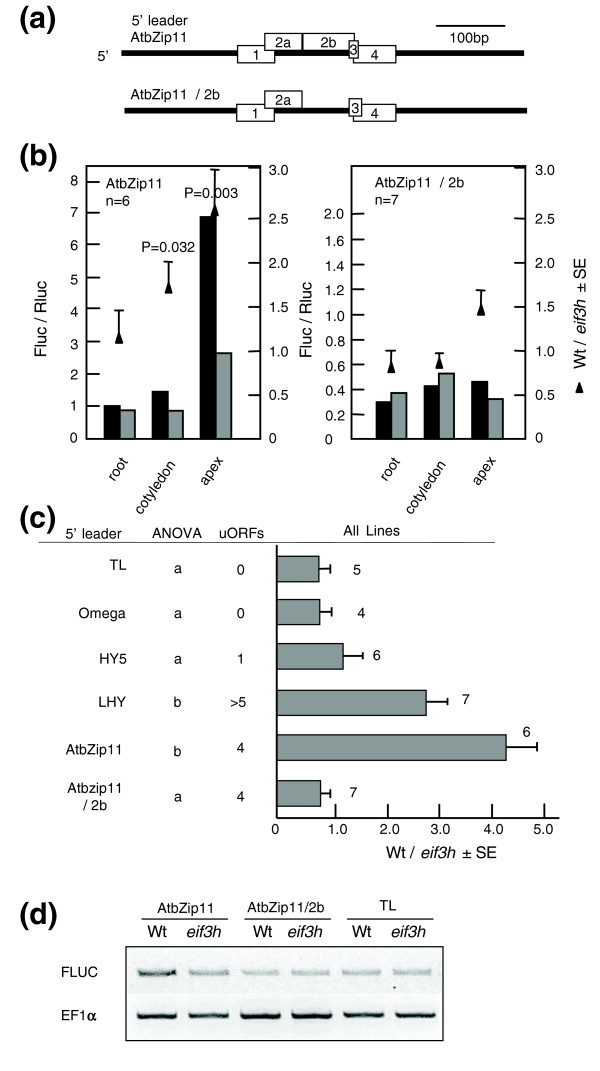
uORF2b contributes to poor translatability of the AtbZip11 leader in the *eif3h *mutant. **(a) **Schematic of the arrangement of uORFs in the AtbZip11 leader. uORF2b was mutated by changing its start codon into a stop codon. **(b) **Translation efficiencies of the AtbZip11 leader lacking uORF2b in three different organs of two-week-old seedlings. The number of lines examined is indicated (n), as are *p *values derived from pairwise *t*-tests. For details see legend to Figure 1. **(c) **Summary of transgenic reporter gene translation assays on six different leader sequences. The number of transgenic lines examined is indicated for each leader, as is the number of uORFs per leader. The letters a and b indicate homogeneous subsets as determined by ANOVA/Tukey test. Thus, leaders that do not share the same letter (a, b) differ significantly in their dependence on the eIF3h protein. SE, standard error. **(d) **Reverse-transcriptase PCR analysis of FLUC mRNA levels in representative transgenic lines harboring TL-FLUC, AtbZip11-FLUC or AtbZip11/2b-FLUC transgenes. The EF1α mRNA was analyzed as a control for equal mRNA levels. The ethidium-bromide stained gels shown here are consistent with other repeat experiments performed with other subsaturating numbers of PCR cycles.

Four other 5' leader sequences were examined for their dependence on eIF3h. Neither the omega leader of tobacco mosaic virus nor the leader of the bZip transcription factor, HY5, was affected by the *eif3h-1 *mutation (Figure 2c and data not shown). Concerning the third example, the leader of tobacco etch virus (TL), one might not expect any difference in gene expression on theoretical grounds, because both Fluc and Rluc are preceded by the same promoter and leader in this case. However, a difference would arise if the mutation in *eif3h *caused differential effects on Fluc and Rluc protein stability, activity, or mRNA levels. The absence of a difference argues against such effects and in favor of the notion that the reporter genes serve as reliable reporters of translation initiation (Figure 1d). As a fourth example, we tested the leader of the LHY myb domain transcription factor [29], which, similar to AtbZip11, harbors multiple upstream open reading frames. The LHY leader did show a tendency for reduced translation in the *eif3h *mutant (Figure 1e), as expected [19].

Within the AtbZip11 leader, the uORF2b is responsible for translational repression in response to sucrose [27]. Eliminating uORF2b from the AtbZip11 leader by mutating its start codon into a stop codon also caused a substantial 'recovery' of translation in the *eif3h *mutant (Figure 2a,b). In actual terms, mutating uORF2b caused a reduction of the Fluc to Rluc ratio in the wild type, perhaps because uORF2b overlaps uORF3 and uORF4 and thus tempers their potentially inhibitory effect on Fluc expression.

Some uORFs have posttranscriptional effects on mRNA stability and mRNA levels, [30-32]. As a first step to address the extent to which eIF3h may affect mRNA levels we examined FLUC mRNA levels in wild-type and *eif3h *mutant seedlings using RT-PCR. As shown in Figure 2d two representative transgenic lines carrying the TL leader or the AtbZip11/2b leader showed approximately equal mRNA levels between wild type and mutant. In contrast, with the original AtbZip11 leader the mRNA level was slightly reduced in the *eif3h *mutant compared to *eIF3h*^+ ^wild-type plants, although the reduction was insufficient to fully account for the difference in FLUC enzyme activity (6.6-fold in this line). These results are consistent with the notion that the lack of eIF3h causes a reduction in translatability of the mRNA as well as a reduction in the mRNA level, possibly by allowing the uORF-containing mRNA to be destabilized.

Although eIF3h protein is expressed in different organs [19], the requirement for eIF3h was most pronounced in the shoot apex and less so, yet still significant, in the cotyledon/hypocotyl (Figure 2b), while in the root, no effect of the *eif3h *mutation could be discerned. The AtbZip11 leader lacking uORF2b showed no dependence on eIF3h in any organ.

In summary, the two leaders tested that harbor multiple uORFs, that is, AtbZip11 and LHY, showed a dependence on eIF3h, while leaders with only one uORF (HY5) showed a marginal and variable dependence on eIF3h, whereas leaders lacking uORFs (TMV omega and the TEV leader (TL)) were not dependent on eIF3h. Despite the evident correlation between uORFs and the requirement for eIF3h, one leader, AtbZip11 with the uORF2b mutation, behaved like an exception in this assay, given that this leader retains four uAUGs. It is plausible that the overall configuration and length of the uORFs, not simply the sheer number alone, defines whether intact eIF3h is needed for optimal expression.

### Microarray experiments

**Figure 3:**
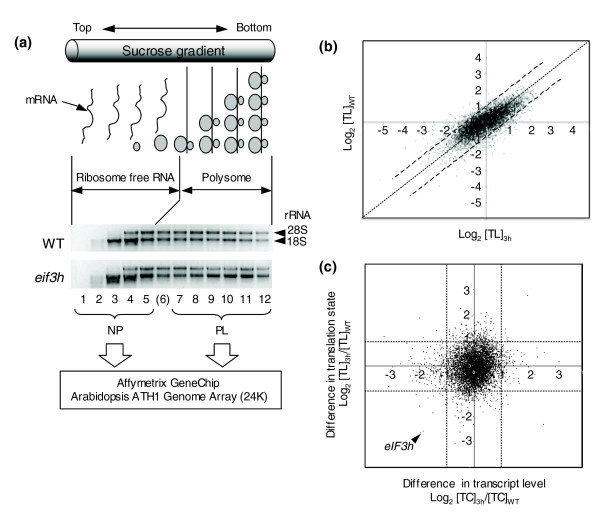
Microarray analysis of polysome loading in the *eif3h *mutant. **(a) **Experimental design for the isolation of polysomal (PL) and non-polysomal (NP) RNAs. After sucrose density gradient centrifugation, samples were collected into 12 fractions. The integrity of the density gradient was confirmed by agarose gel electrophoresis and visualization of ribosomal RNAs with ethidium bromide. Microarray probes were generated from pooled samples as indicated. **(b) **Log_2 _transformed average translation states (TL = PL/NP) of the *eif3h *mutant were plotted against the data from wild-type plants. **(c) **Effects on polysome loading by the *eif3h *mutation (Log_2 _[TL]_3h_/[TL]_WT_) were not generally correlated with effects on transcript levels (Log_2 _[TC]_3h_/[TC]_WT_). An arbitrary two-fold cut-off was applied to highlight responsive genes (dotted lines). The number of genes affected both transcriptionally and translationally is very small (25 out of 6,238 genes for which reproducible data were available). Among them, the *eIF3h *mRNA is indicated by an arrow head.

To examine whether there exists a general requirement for eIF3h for efficient translation of mRNAs harboring uORFs, microarray analysis was carried out using polysomal (PL) and non-polysomal (NP) RNA samples collected by sucrose density gradient centrifugation from *eif3h-1 *mutant and wild-type plants (Figure 3a). Total RNA samples were also isolated to monitor the effect of the *eif3h *mutation on mRNA transcript (TC) levels. Labeled samples were hybridized to *Arabidopsis *Affymetrix ATH1 GeneChip arrays (approximately 24,000 genes) and the resulting signals were normalized as described in the Materials and methods. Hybridization signals from each array are routinely adjusted to the same total intensity to compensate for differences in labeling and hybridization efficiency. Therefore, mRNAs that are translationally inhibited more than the average mRNA by the *eif3h *mutation will appear as undertranslated, and vice versa. In any event, the ratio of total polysomal/non-polysomal RNA was similar between *eif3h *mutant and wild type (Figure 3a) [19]. Thus, if the normalization procedure did mask a global shift in polysome loading, this shift must have been minor or negligible.

The 8,831 genes showing 'present' or 'marginal' expression across all 12 arrays, including two biological repeats, were considered for further analysis, whereas genes scored as 'absent' were excluded (see Additional data file 1 for scatter plots).

In the following, the term 'translation state' [TL] designates the ratio of the signal intensity between polysomal and non-polysomal samples (TL = PL/NP). Expressed as log_2 _transformed data, a positive value indicates that more transcripts were associated with ribosomes, and a negative value indicates that more transcripts were in a ribosome-free state. Both in wild-type and mutant plants, the mRNA translation state ranged from highly polysomal to highly non-polysomal, over approximately a 64-fold range (Figure 3b).

Next, comparisons of the translation states of *eif3h *mutant and wild-type plants were performed by calculating [TL]_3h_/[TL]_WT_. After log-transformation for ease of display, a positive value indicates that an mRNA is more polysomal in the *eif3h *mutant than in the wild type, and vice versa. The difference in total mRNA transcript level was expressed using a simple log_2 _transformed ratio of [TC]_3h_/[TC]_WT_. Among 6,854 genes that yielded reproducible polysome loading data (see Materials and methods for selection criteria), 246 genes were translationally inhibited in the *eif3h *mutant, based on an arbitrary two-fold cutoff, and 188 genes were translationally stimulated (Figure 3c; see Additional data file 2 for gene lists). Changes in the transcript level were not obviously correlated with changes in translation state (Figure 3c). Exceptionally, the *eIF3h *gene itself was clearly suppressed at both the translational and transcriptional levels, presumably a consequence of the T-DNA insertion in the 10th exon of the gene. This result is consistent with the low level of the truncated eIF3h protein detected in the *eif3h-1 *allele [19]. The defects in the *eif3h-1 *mutant may be a consequence of both the reduced expression level and the truncation of the carboxyl terminus. The general trends of the microarray-based differences in translation states and transcript levels were reproduced by quantitative real-time PCR amplification using 13 different genes (Additional data file 3).

### Functional classes of genes misregulated in the *eif3h-1 *mutant

**Figure 4:**
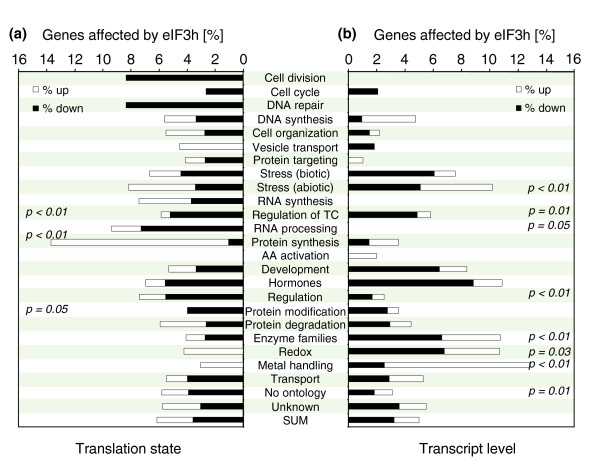
Survey of trends in translational stimulation and repression among functional classes of genes. The changes in **(a) **translation states or **(b) **transcript level observed between wild type and *eif3h *are shown after gene ontology analysis using MapMan v1.8.0 [33]. Bars represent the percentage of responsive genes in a particular class when a two-fold cut-off was applied. *X*^2 ^tests were carried out to evaluate the extent of deviation from the average pattern and *p *values are given.

**Figure 5:**
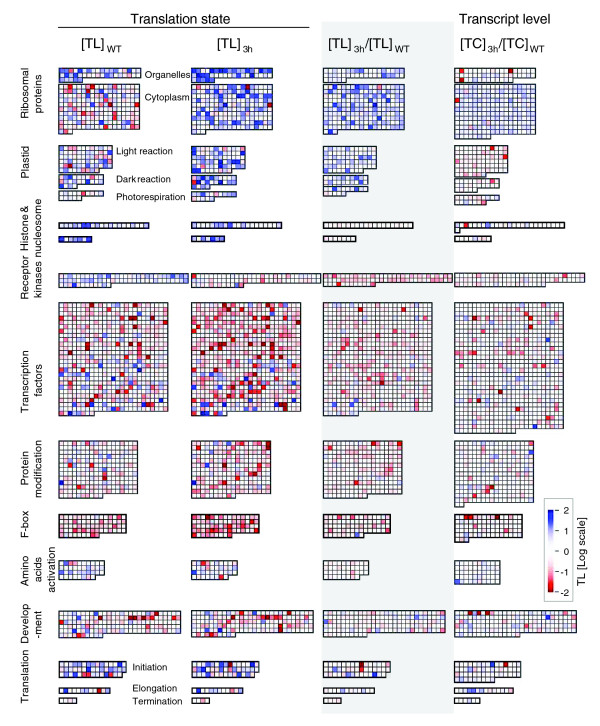
Certain functional classes of mRNAs show a coordinated translational response to the *eif3h *mutation. Microarray data were plotted onto *Arabidopsis *biochemical pathways and functional categories using MapMan v1.8.0. Each square represents a single gene. On the log color scale, light blue refers to a 2-fold (log_2 _= 1) stimulation of polysome loading or transcript level in the *eif3h *mutant compared to wild type. Note the translational stimulation of ribosomal proteins and plastid proteins in the *eif3h *mutant and the translational reduction for receptor kinases, transcription factors, F-box proteins, and protein modifying enzymes. Other classes are shown as non-significant controls.

To examine whether or not genes that were mistranslated in the *eif3h *mutant fall into specific functional groups, the microarray datasets were fed into MapMan (v1.8.0 [cell_functions_overview]) [33], which projects data from *Arabidopsis *Affymetrix arrays onto diagrams of metabolic pathways and gene ontology classes (Figures 4 and 5). One group of genes was biased toward translational stimulation in the *eif3h *mutant, namely protein synthesis related genes (*p *< 0.01; *X *^2^-test), in particular cytosolic proteins for small and large ribosomal proteins, but also organellar ones (Figures 4 and 5). Interestingly, with few exceptions (eIF3g1, eIF3k and nCBP [novel cap-binding protein]), the mRNAs for translation initiation factors did not partake in the translational stimulation, nor did other core 'protein synthesis' mRNAs, such as those for aminoacylation, translation elongation or termination (Figure 5, bottom).

A higher resolution classification using MapMan revealed an additional functional group with a coordinated trend for translational enhancement in the *eif3h *mutant, namely cytosolic mRNAs encoding photosynthesis-related proteins in the chloroplast (Figure 5, top). Overall, among the 188 translationally upregulated genes, 24.3% were protein synthesis related, and 6.6% were related to photosynthetic light and dark reactions. For comparison, although many histone and nucleosome assembly related genes were highly polysomal in the *eif3h *mutant, they were also highly polysomal in wild type, resulting in a largely unchanged translation state (Figure 5).

A statistically significant bias toward translational inhibition in the *eif3h *mutant could be seen for genes annotated as transcriptional regulators and protein modifiers (Figure 4a). A higher resolution classification revealed that transcription factors had variable polysome loading in the wild type; whereas receptor kinases, which were the most strikingly downregulated group, generally dropped from a highly loaded state in the wild type to a medium level in the mutant (Figure 5). In contrast, many other metabolic pathways represented in MapMan were not coordinately affected by the *eif3h *mutation, for example, development, cell wall synthesis, the tricarboxylic acid cycle, and lipid, amino acid, secondary, nitrate, and sulfate metabolism (Figure 5 and data not shown). Taken together, these results clearly suggest that certain functional classes of mRNAs share specific features that make them dependent on the activity of eIF3h in a coordinated fashion.

### Analysis of *Arabidopsis *5' untranslated region sequences

Previous results indicated that the eIF3h protein plays a role in overcoming the inhibitory effects on ribosome scanning and translation initiation caused by uORFs (Figures 1 and 2) [19]. Because reduced translation initiation due to uORFs is reflected in reduced polysome loading [20], we carried out a series of computational analyses on the polysome microarray datasets to further test and extend this hypothesis.

**Figure 6:**
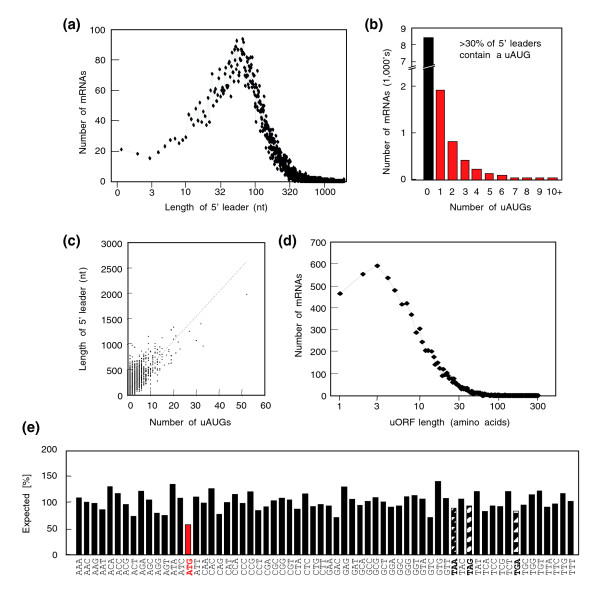
Characterization of *Arabidopsis *5' leader sequences. The analysis is based on a set of sequences obtained from cap-purified mRNAs (see Materials and methods). **(a) **Length distribution. **(b) **Number of uAUGs. **(c) **Correlation between length of the leader and number of uAUGs. **(d) **Distribution of uORF lengths among the 12,129 *bona fide *full-length leader sequences. uORFs that overlap the main ORF were not included in this analysis. **(e) **The frequency of each dinucleotide (AA, AC, and so on) was determined empiricially across all 5' leaders (not shown). Then, the theoretical frequency of each triplet was predicted based on the dinucleotide data (see Materials and methods for details) and set to 100%. The empirical frequency of each triplet across all 5' leaders was then expressed in relation to the predicted frequency.

First, the entire set of *Arabidopsis *5' mRNA leader sequences based on the longest expressed sequence tag sequences were downloaded from the *Arabidopsis *Information Resource (TAIR). Since these may contain partial sequences, only the 5' leaders of genes listed in the SSP (Salk/Stanford/plant gene expression center) consortium's full-length cDNA list [34] (March 2006) were extracted, and the resulting 12,129 full-length transcript sequences were used for further analysis. The average 5' leader length was 131 bases. With the exception of leaders shorter than 20 nucleotides (nt), the distribution of the log-transformed leader lengths approximately matched a normal distribution, with a geometric mean of 91 (Figure 6a). Among the full-length transcripts, 3,735 (30.8%) contained at least one uAUG in their 5' leader (Figure 6b; Additional data file 4), which is higher than previous estimates (22% of 1,023 *Arabidopsis *genes [35]). The number of uAUGs correlated roughly with the length of the 5' leader sequences (Figure 6c). Figure 6d shows the distribution of uORF length. The AUG triplet is the most underrepresented triplet in 5' leaders, indicating a bias against translational start codons, but surprisingly its frequency was only two-fold lower than expected by chance alone (Figure 6e; see Materials and methods for details). No such bias was detected in the 3' untranslated regions (not shown). Using similar criteria, we examined the frequency of the AUG triplet in positions that result in uORFs overlapping the main ORF. Even in these positions, which must be considered strongly inhibitory for translation of the main ORF, the AUG triplet was underrepresented only between two and threefold (not shown).

Among the 30% of genes containing uORFs, almost half (1,602 or 13.2% of all mRNAs) have at least one AUG in a favorable context for plants (AnnAUGn or GnnAUGG) [36-38]. Thus, many of the uAUGs are expected to be recognized by the scanning 40S subunit, rather than bypassed by leaky scanning. Moreover, 12.9% of all uAUGs (1,135 out of 8,783) either initiate, or are part of, a uORF that overlaps the main ORF (data not shown). Of these, one third (346 or 30.4%) were in a favorable start codon context. Taken together, these analyses reveal an abundance of *bona fide *translated uORFs in 5' leaders of *Arabidopsis *mRNAs whose sequence has been experimentally validated.

### Sequence features of translationally regulated genes

Next, we asked whether the eIF3h-dependence of a given transcript (Log_2 _[TL]_3h_/[TL]_WT_) could be explained by features extracted from the 5' leader sequence. A recent large-scale analysis of *Arabidopsis *transcripts [39] addressed the level of variation among transcripts from the same gene. Where alternative transcription start sites exist, they are usually less than 10 bases apart and when they do occur in the 5' leader they usually consist of small shifts in splice acceptor or donor sites of typically far fewer than 30 bases. Therefore, using a single full-length cDNA sequence to search for signals affecting polysome loading is an acceptable simplification.

**Figure 7:**
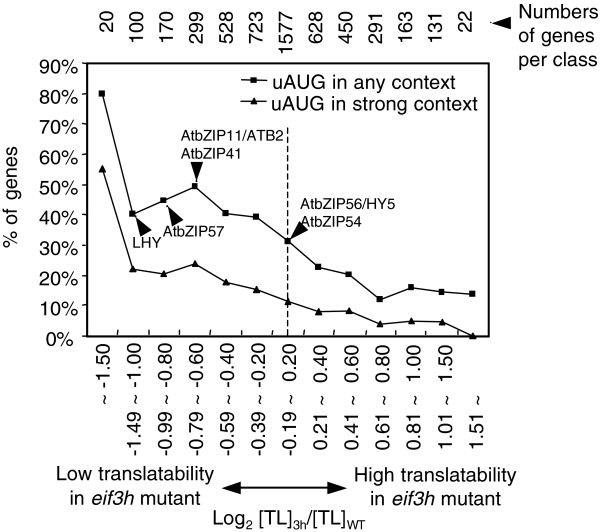
eIF3h is responsible for enhancing the translation state of mRNAs harboring upstream AUGs. mRNAs were classified into bins according to the difference in translation state between *eif3h *mutant and wild type (log_2 _[TL]_3h_/[TL]_WT_). The proportion of genes containing uAUGs was determined for each bin. 'Strong context' refers to uAUGs in the sequence context AnnAUGn or GnnAUGG, which are close matches to the optimal Kozak consensus for translation initiation.

As we hypothesized, gene sets that were translationally repressed in the *eif3h *mutant contained a high proportion of genes harboring uAUGs (Figure 7). In detail, 80% of all mRNAs in the most strongly eIF3h-dependent class contained at least one uAUG. Most of these transcripts (55%) had at least one uAUG in a strong context. These uORFs generally do not overlap the main ORF but terminate within the 5' leader (not shown). By contrast, the transcripts that were translationally stimulated in the mutant were far less likely to harbor uAUGs; down to 14% in any context and down to 0% when only strong uAUGs were considered. These significant deviations from the average abundance of uAUGs clearly suggest that eIF3h is needed, transcriptome-wide, for the efficient translation initiation on mRNAs that contain uAUGs, although other factors must contribute. Among the translationally compromised genes were LHY and AtbZip11, consistent with earlier observations (Figures 1 and 2). In addition, AtbZip41 and AtbZip57, two other mRNAs with similar uORF patterns as AtbZip11 [27,28] were also found in the undertranslated set (Figure 7), whereas HY5, a bZip factor with a single uORF that was not translationally affected in the reporter gene assay (Figure 2c), was also not affected according to the microarray. The extent of the reduction in polysome loading in the *eif3h *mutant was less than expected from the reporter assays (Figures 1 and 2); this may be due to the fact that the reporter assay measures the compounded effects of mRNA stability and translatability whereas the microarray measures translation state as indicated by polysome loading and is not confounded by mRNA levels.

Because the eIF3h-dependent genes tend to cluster according to functional categories and tend to contain uORFs, we predicted that categories of genes that are enriched in uORFs might be particularly dependent on eIF3h in their ribosome loading and vice versa. The percentage of genes harboring uORFs in each of MapMan's 'cellular function' categories varied widely (Table 1), from 11.5% in the protein synthesis category all the way up to 39.5%, 40.5%, and 52.5% for the categories transcriptional regulation, cell division, and protein modification, respectively. Incidentally, uORFs are also enriched among proto-oncogenes and genes functioning in cell growth and transcriptional regulation in mammalian genomes [40].

**Figure 8:**
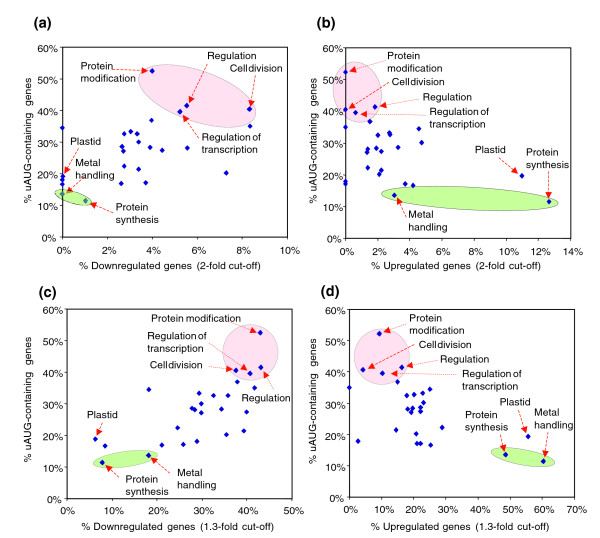
uORF content and eIF3h-dependent polysome loading are correlated across functional categories of genes. Each datapoint represents a functional class of genes. The percentages of translationally up- or downregulated genes were plotted against the percentages of uAUG-containing genes in a given functional class. The four classes with the highest occurrence of uORFs and the two classes with the lowest occurrence are highlighted with pink and green circles, respectively. The left panels **(a, c) **focus on genes whose polysome loading is reduced in the *eif3h *mutant; whereas the right panels **(b, d) **focus on genes with stimulation of polysome loading. **(a, b) **Stringent cutoff; only genes with a two-fold or higher difference in polysome loading were considered. **(c, d) **Relaxed cutoff; genes with a 1.3-fold or higher difference in polysome loading were considered. Note that the correlation is apparent in each case.

When the percentage of eIF3h-dependent genes was plotted against the percentage of uORF containing genes, a clear correlation emerged across all 26 functional categories (Figure 8a,c), regardless of the precise cutoff value to define the downregulation in polysome loading. Vice versa, groups of genes enriched in uAUGs tended to contain a very low percentage of genes that were upregulated in the *eif3h *mutant (Figure 8b,d). This correlation underscores the role of eIF3h in the polysome loading state of uORF-containing mRNAs.

**Figure 9:**
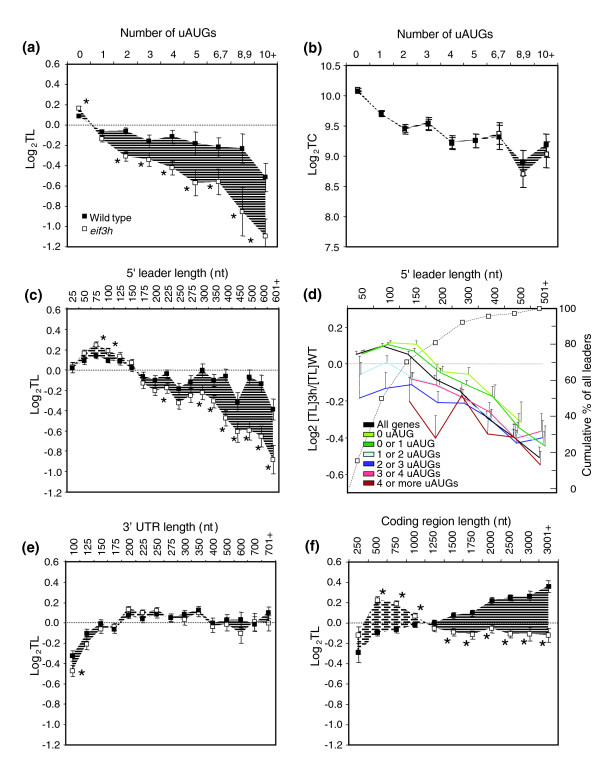
Identification of structural parameters that predict mRNA polysome loading in wild type and *eif3h *mutants. The average translation state of mRNAs was plotted separately for wild-type and *eif3h *mutant plants after subdividing the transcriptome (5,101 genes with polysome loading data and full-length cDNA support) according to the following parameters: **(a) **number of uAUGs in the 5' leader; **(b) **the *eif3h *mutation did not cause a global reduction in mRNA transcript levels for uORF-containing mRNAs; **(c) **length of the 5' leader in nucleotides (nt); **(d) **similar to (c), but leaders were classified into subgroups according to the number of uORFs. For clarity, we plotted only the difference in translation state between *eif3h *and wild type (colored lines). The stippled line denotes the percentage of leaders in each length class (cumulative). **(e) **Length of the 3' untranslated region. **(f) **Length of the protein-coding region of the main ORF. Standard errors are shown. The asterisk indicates a significant difference between the translation state in wild type and *eif3h *mutant (*p *< 0.05) according to a *t*-test (unpaired, two-tail).

Because the correlation between uAUGs and eIF3h-dependent translation (Figure 7) was incomplete, there must be factors other than uAUGs that influence the polysome loading state in the *eif3h *mutant. Consistent with earlier analyses, Figure 9a shows that increasing numbers of uAUGs were more inhibitory to the translation state [TL] in the *eif3h *mutant than in the wild type; however, presence of uAUGs did not generally result in a lower level of total mRNA (Figure 9b). Because the likelihood of uAUGs increases with the length of the 5' leader (Figure 6c), it was expected that long leaders would be preferentially undertranslated in the *eif3h *mutant, and they were (Figure 9c). However, length of the leader plays a more profound role because it contributed to eIF3h-dependence regardless of the number of uORFs and even in the absence of uORFs (Figure 9d). The effect of uORFs was most striking among 5' leaders up to 300 nt long, which make up 92.4% of the genes. The effect of leader length may be partly due to translation initiation at non-AUG codons, which does occur in *Arabidopsis *[41], although we do not rule out other explanations.

The length of the main coding region also affected polysome loading in a manner sensitive to eIF3h, while the length of the 3' untranslated region (UTR) did not have any effect (Figure 9e,f). In the wild type, the longer the main ORF, the higher the translation state. This was expected, given that longer mRNAs have room for more ribosomes. Surprisingly, the opposite effect was seen in the mutant; that is, longer main ORFs were significantly more dependent on eIF3h than main ORFs of intermediate length. Although leader length and main ORF length may cause eIF3h-dependence by the same mechanism, we first considered these variables separately. Main ORF length and uORF number affected the requirement for eIF3h independently because the effect of main ORF length could be seen among mRNAs lacking uORFs, there was no correlation between main ORF length and leader length or number of uORFs, and the inhibitory effect of uORFs was retained in mRNAs with main ORF lengths between 750 and 1,500 nt (not shown).

**Figure 10:**
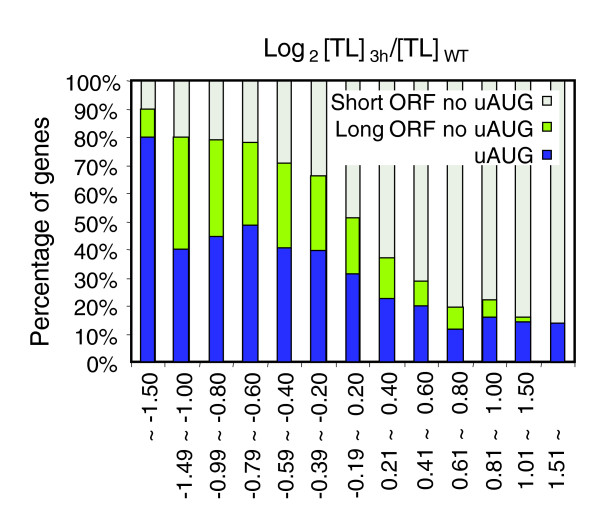
Contribution of the length of the main ORF versus the presence of uORFs to the translation state of mRNAs. Bins selected according to the extent of eIF3h-dependence (Figure 7) were examined for the percentage of genes with a long (> 1,300 nt) main ORF but no uAUG, a short main ORF (< 1,300 nt) and no uAUG, and any number of uAUGs.

Together, presence of uORFs and length of the main ORF appear to be responsible for the majority of cases where translation state is dependent on eIF3h (Figure 10). uORFs are present at high frequencies in genes for transcription factors and protein modifying proteins. Accordingly, these classes of genes are among the most dependent on eIF3h function. Among the mRNAs least dependent on eIF3h for high polysome loading are those coding for ribosomal proteins.

## Discussion

The eIF3 h subunit (eIF3h) is one of the non-core subunits of the eIF3 protein complex. eIF3 subunits h and e in particular have emerged as candidate genes during tumorigenesis, by being overexpressed [42], or because expression of intact or truncated forms can induce tumor formation [43-45]. By dissecting the precise contributions of these proteins to translation initiation their role in tumor formation may become clearer. Our previous data from transient reporter gene expression assays suggested that mutation of eIF3h compromises translation of a subset of transcripts [19]. Here we first extended these findings to stable transgenic plants (Figures 1 and 2). The microarray data then highlighted that eIF3h is responsible genome-wide for the efficient polysome loading of mRNAs carrying uORFs and also seems to preferentially boost the translation state of long mRNAs.

Among the factors in the 5' leader sequence that affect the mRNA translation state are length [46] and uORFs [25]. To analyze the effects of these factors, we used the 5' leader sequences of full length *Arabidopsis *mRNAs known to be purified by means of 5' cap binding [47]. The evidence that the effects on translation states measured using microarrays are reliable and significant is as follows. First of all, chance alone does not explain correlations in translation state across gene ontology classes (Figure 5) and would not result in the coordinated changes in translation state observed, for example, for ribosomal proteins. Moreover, the fact that the majority of the variation in eIF3h dependence between different mRNAs can be attributed to just three factors, uORFs and length of the main ORF or 5' leader (Figures 9a,d,f and 10), also speaks for the authenticity of the data. Furthermore, the general tendencies in translation state deduced from the microarray analysis matched the trends from reporter gene expression assays (Figures 1, 2, and 7).

### Translational regulons

In mammalian cells, uORFs are enriched among mRNAs for regulatory proteins, including transcription factors, receptor proteins, signal transduction components, and proto-oncogenes [40]. This notion is borne out by the small set of plant mRNAs with uORFs that have been studied (for example, [21,26,27,48]) and is echoed by this analysis (Figure 8, Table 1). The altered translation state of many important regulatory proteins may have contributed to the substantial effects on mRNA transcript levels in the *eif3h *mutant (Figure 3) and may ultimately underlie the pleiotropic phenotype of the *eif3h *mutant [19].

One class of mRNAs, coding for ribosomal proteins, showed widespread increases in polysome loading in the *eif3h *mutant (Figure 5). A coordinated reduction of polysome loading for ribosomal protein mRNAs has been reported earlier in yeast (by the nonfermentable carbon source glycerol [49], and amino acid starvation [50]) and in mammalian cells (by dexamethasone [51,52]). Are there clusters of mRNAs whose translation is regulated in a coordinated fashion in response to a number of different stimuli? Such a phenomenon would be indicative of a regulon of translational control, a concept whose biological utility is not yet widely established. The *Arabidopsis *ribosomal proteins show tight translational co-regulation in response to drought stress and hypoxia [53,54], and also respond as a cohort to a mutation in *eIF3h*. Together with a prior study in *Caenorhabditis elegans *[55], this finding exemplifies that mutations in a *bona fide *generic translation factor can uncover a coordinated translational response suggestive of a translational regulon. Interestingly, the mRNAs for mammalian translation initiation factors are regulated transcriptionally (by glucocorticoids) rather than translationally [52], a finding echoed by the lack of translational coordination between ribosomal proteins and translation initiation factors observed here (Figure 5).

The translational co-regulation of ribosomal protein genes may reflect a coordinated attempt of the cell to compensate for the deficiency of eIF3h. However, the potential sensing and response mechanisms are unknown. Other than their mammalian counterparts [56], *Arabidopsis *ribosomal protein genes generally do not share canonical 5' terminal oligopyrimidine tracts (5' TOP motifs, not shown). Ribosomal protein mRNAs generally have short main ORFs, and their 5' leaders tend to be devoid of uORFs (Table 1), short, and GC-rich [53]. However, these features do not explain the coordinated upregulation of translation state in the *eif3h *mutant, because a random collection of mRNAs with these features did not show such a uniform response (data not shown).

### The function of eIF3h

Our results suggest that eIF3h contributes to polysome loading in at least two major ways. First, eIF3h mitigates the repressive effect of uORFs (for example, Figure 9a). This repression is associated with, but not explained by, a repression of mRNAs with long 5' leaders. Secondly, and unexpectedly, long main ORFs appear to be particularly dependent on intact eIF3h (Figure 9f). uORF number and leader length co-vary, but the effects were separable (Figure 9d). uORFs and length of the main ORF are not correlated and, therefore, the two effects are independent. Thus, having a long main ORF and a leader with uORFs is a predictor for eIF3h-dependent translation, whereas having a short main ORF and no uORFs is a predictor for eIF3h-independent translation (Figure 10). At this point it is useful to consider length of the main ORF and length of the 5' leader together. A role for eIF3h as an initiation factor is more easily reconciled with its effect on uORF-containing mRNAs than on mRNAs that are simply long. Is the effect of length an indirect one? Although this cannot be ruled out, we note that the gene lists for altered polysome state or transcript level include very few initiation or elongation factors aside from *eIF3h *itself (Figure 5 and Additional data file 2). Moreover, if elongation was slowed down in *eif3h*, one would expect elevated polysome loading in the mutant, exactly the opposite of what was observed. The effect of mRNA length may, however, be an indirect consequence of competition between the relatively abundant, yet short, mRNAs for ribosomal and plastid proteins, which retain high polysome loading, and other, longer mRNAs. On the other hand, it may also point toward more direct, though entirely speculative, roles of eIF3h in the translation initiation of long mRNAs. For example, it is not difficult to imagine that long and short mRNAs may differ in their tendency for circularization into a closed loop via poly(A)-binding protein [57,58], and also that closed-loop re-initiation and *de novo *initiation may differ in their requirement for initiation factors such as eIF3h. Distinguishing between this and other plausible explanations, such as increased ribosome dropoff during scanning or elongation, will require additional experiments.

Translational regulation by uORFs occurs in a number of different ways (reviewed in [25]). In the leaky scanning model, some of the scanning ribosomes recognize the uAUGs as a functional start codon, thereby reducing the chance to start at the main ORF, but some can pass the uAUG without initiation and thus reach the main ORF [59]. According to the re-initiation model, the ribosome recognizes the uAUG as a start codon, but after termination of the uORF the ribosome resumes scanning until it encounters the main AUG codon. Thereby the efficiency of re-initiation can control the efficiency of initiation at the main AUG [60].

The eIF3 complex has only recently been implicated as a regulator at or around a uORF [17-19]. Generally, eIF3 prevents premature association of 40S and 60S ribosomal subunits, promotes the association of ternary complex and 40S ribosomal subunit (43S complex formation), functions as a scaffold for other initiation factors, and stimulates the binding of mRNA to the 43S pre-initiation complex (48S complex formation; reviewed in [3-5]). Yet, the *eif3h *mutations used here do not seem to affect global translation initiation [19]. Our results suggest that eIF3h may contribute to functions of eIF3 downstream of 48S pre-initiation complex formation. Potential roles are in scanning processivity by the 40S, selection of the initiation codon, or in the resumption of scanning or re-initiation downstream of a uORF. It is also possible that a primary defect in translation initiation in the *eif3h *mutant will have secondary downstream effects, which may include destabilization of a uORF-containing mRNAs. However, mRNAs identified as eIF3h-dependent in the microarray did not generally have lower mRNA transcript levels (Figures 3c and 9b). To date, we have not detected any association between translation state in *eif3h *and initiation codon context. A defect in scanning processivity would predict a correlation between the length of the leader and the requirement for eIF3h, and such a correlation was observed (Figure 9d). Some uORFs inhibit initiation in a fashion dependent on their coding sequence, more often by peptide-dependent stalling of the ribosomes than by rare codons [26,61,62]. However, the lengths and sequences of uORF peptides of eIF3h-dependent genes are very diverse (data not shown), arguing that the *eif3h *mutation does not cause peptide sequence dependent stalling of ribosomes within uORFs. Sequence-independent uORFs are found in yeast GCN4, other transcription factor mRNAs and elsewhere [21,63] (reviewed in [24]). They are inhibitory to translation because resumption of scanning and acquisition of a new ternary complex are considered inherently inefficient, compared with the 43S loading of an mRNA at the 5' cap. The cumulative inhibition by multiple uORFs in the *eif3h *mutant suggest that re-initiation following translation of a uORF may be the process in which eIF3h plays a major role.

The molecular mechanism of re-initiation is not clear, but it must involve a decision by the 40S subunit whether to resume scanning or not, and then the scanning ribosome needs to be replenished with a new ternary complex for the next initiation attempt. Re-initiation becomes less efficient as uORFs get longer [22,64] but more efficient when the uORF is followed by a long intercistronic spacer sequence [65]. Interestingly, the defect in the *eif3h *mutant was not exacerbated by uORF length, at least not for single uORFs, and was not mitigated by a long spacer (data not shown). It is also known that re-initiation appears to be more efficient when the uORF is first recognized in a cap-dependent fashion and with the full complement of initiation factors than when the uORF is recognized via an internal ribosome entry site that allows initiation without eIFs [66]. These results suggest that the eIF-primed ribosome has an inherent competence to resume scanning after termination of a uORF, but its competence is gradually lost during peptide elongation. Although there is evidence that the initiation factors are lost when a scanning ribosome begins to synthesize a polypeptide from a uORF, it is not clear whether all the initiation factors are lost, nor whether the loss occurs immediately after the initiation or after some time of elongation [22]. Additional experiments are needed to define more precisely whether or how eIF3h contributes to re-initiation.

## Conclusion

Taken together, all these observations suggest that eIF3h functions in translation initiation by overcoming the repressive effect of uORFs, and by boosting the polysome loading of mRNAs with a long leader or long main ORF. Independent confirmation of the data from polysome microarray experiments was provided by translational reporter gene expression cassettes introduced into stable transgenic plants. The exact mechanism of eIF3h's activity remains to be further defined; neither do we rule out that eIF3h may play additional roles in translation initiation or beyond. It is noteworthy, however, that long uORFs such as those found to confer eIF3h dependence in AtbZip11 appear to be highly inhibitory in budding yeast [64], a species that does not possess a recognizable ortholog of eIF3h. uORFs are particularly abundant among *Arabidopsis *mRNAs encoding regulatory proteins such as transcription factors and protein kinases. Given the growing appreciation for uORFs as modules of translational control, eIF3h may be regarded as a cog in the machinery of translational regulation.

## Materials and methods

### Molecular cloning procedures

A *Renilla *luciferase reporter gene was inserted into the binary T-DNA vector pFGC19, which shares the vector backbone and Basta resistance gene with pFGC5941 (kindly provided by R Jorgensen). Transcription units consisting of selected 5' leader sequences and the firefly luciferase coding region under the control of cauliflower mosaic virus 35S promoter and terminator sequences [19] were spliced as *Hin*dIII fragments to reside adjacent to the Rluc reference gene. Each T-DNA was transferred to *A. thaliana *ecotype Wassilewskija by floral dip transformation of plants heterozygous for the *eif3h-1 *mutation. T1 transgenic plants were selected on Basta (5 mg/l) and selfed to derive T2 families. Gene expression assays were conducted in T2 seedlings or, after additional selfing, in the T3.

### Assays of translation in transgenic plants

*Arabidopsis *seedlings were grown on agar-solidified (0.8%) half-strength Murashige and Skoog salts (pH 5.7) containing 1% sucrose in constant light for 10-12 days, if not stated otherwise. *eif3h *mutant seedlings were identified among the wild-type siblings by visual inspection. Between three to seven *eif3h *seedlings and five wild-type seedlings were picked, the roots were cut off and the pooled shoots were subjected to the dual luciferase assay, essentially as described by the supplier (Promega, Madison, WI, USA). For each transgenic line, the experiment was repeated between four and ten times on different days. For each construct, multiple lines, typically five or six, were recovered and examined. The ratio between firefly luciferase and *Renilla *luciferase activity is regarded as one raw data point. It reflects the relative efficiency of gene expression on the 5' leader being tested in comparison with the tobacco etch virus leader (TL), which precedes the *Renilla *luciferase reference gene. The Fluc/Rluc ratios were log-transformed. The effect of the *eif3h *mutation on translational activity was determined by calculating the fold-difference of the Fluc/Rluc ratio between wild-type and *eif3h *mutant siblings (WT/*eif3h*). These were averaged from multiple biological repeats and displayed with their standard error (SE). The potential difference in Fluc/Rluc ratios between wild-type and *eif3h *mutant plants was statistically evaluated by a two-tailed paired Student's *t*-test using Microsoft Excel software. Analysis of variance with a Tukey *post-hoc *test was conducted to distinguish eIF3h-dependent from eIF3h-independent leader sequences.

### Plant growth and RNA sample preparation for microarray

*A. thaliana *wild-type and *eif3h-1 *mutant seedlings [19]) were grown on agar plates containing full strength Murashige and Skoog salts (pH 5.7) supplemented with 1% sucrose. Stratified seeds were germinated and grown for 10 days under continuous light at 22°C. For harvesting, liquid nitrogen was poured directly onto the plates and the aerial parts of the frozen seedlings (approximately 500 mg) were collected by scraping. Harvested samples were ground in liquid nitrogen with a mortar and pestle and then resuspended in 2 ml of extraction buffer (0.2 M Tris-HCl, pH 8.0, 50 mM KCl, 25 mM MgCl_2_, 2% polyoxyethylene 10 tridecyl ether, 1% deoxycholic acid, 50 μg/ml cycloheximide, 400 U/ml RNasin^® ^(Promega); modified from [67]). After spinning for 5 minutes at 4°C, 1 ml of supernatant was loaded onto a 10 ml continuous sucrose gradient (15% to 50%) in a polyallomer tube and spun in a Beckman SW41Ti rotor at 35,000 rpm for 3.5 h at 4°C. We collected 12 fractions by carefully pipetting 900 μl/fraction from the top, and mixed immediately with 600 μl of phenol:chloroform:isoamylalcohol containing 15 μl of 10% SDS, 12 μl of 0.5 M EDTA, and 3 μl of 0.1 M aurin tricarboxylic acid. After isopropanol precipitation of the soluble fractions the pellets were dissolved in 20 μl of RNase-free water. The quality of polysome isolation was examined by electrophoresis. Based on UV absorption profiles obtained from identical but separate experiments as well as the electrophoretic gel pictures, the polysomal and non-polysomal fractions were determined. Under our conditions, the top five fractions (1-5) contain ribosome-free mRNAs and monosomes and the bottom six fractions (7-12) contain mRNAs associated with multiple ribosomes [19] (Figure 3a). These fractions were pooled together to make non-polysomal (NP) and polysomal (PL) RNA samples. Total RNA was isolated from the aerial part of seedlings using TRI reagent (Sigma, St Louis, MO, USA) by following the manufacturer's guide.

All RNA samples were treated with DNase I before reverse transcription and purified using RNeasy Mini Spin Columns (QIAGEN, Hilden, Germany). For two biological replicates, two independent RNA preparations were carried out on different days.

### Microarray data analyses

The GeneChip *Arabidopsis *ATH1 Genome Array containing approximately 24,000 genes were purchased from Affymetrix (Santa Clara, CA, USA). GeneChips were processed at the University of Tennessee Affymetrix Core Facility. From the isolated total RNA samples first and second strands of cDNA were synthesized. The biotin-labeled cRNA was generated by *in vitro *transcription and hybridized to a GeneChip at 45°C for 16 h. After hybridization, the GeneChip was washed and stained with streptavidin-phycoerythrin (Invitrogen-Molecular Probes, Carlsbad, CA, USA), followed by a wash with biotinylated antibody goat IgG and another staining with streptavidin phycoerythrin. The GeneChips were immediately scanned with a GeneChip 7G high-resolution scanner. The individual GeneChip scans were quality checked for the presence of control genes and background signal values. The data were background-corrected and normalized using the Affymetrix MAS 5.0 software. The genes showing 'present' or 'marginal' calls across all replicate chip data including [TC], [PL], [NP] were considered for further analysis. The translation state [TL] was expressed as the ratio of [PL]/[NP]. To assess the change in translation state in the *eif3h *mutant, the ratio [TL]_3h_/[TL]_WT _was determined. The change in transcript level was expressed by the simple ratio [TC]_3h_/[TC]_WT_. All these values were typically log_2 _transformed for display and statistical analysis.

### Statistical analysis of microarray data

Because most of the subsequent analyses relied in part on data from genes that were not significantly changed by the *eif3h *mutation, we adopted the following filtering procedure [68]. The normalized expression values from the two biological replicates were averaged and standard deviations were calculated. Data that met at least one of the following three criteria were considered as reliable and used for further analyses. First, both of the replicates showed more than a two-fold difference in the same direction (up/down); second, the average value showed more than a two-fold difference AND the standard deviation was less than 50% of the average; or third, the standard deviation was less than 0.5 regardless of the fold change. These datasets thus selected consisted of 6,854 genes for polysome data (PL and NP) and 7,976 genes for transcript data (TC). Within this filtered set of data, 246 genes were translationally inhibited in the *eif3h *mutant, based on an arbitrary two-fold cutoff, and 188 genes were translationally stimulated. At the transcript level 253 and 144 genes were considered down- and upregulated, respectively, in the *eif3h *mutant. To validate this approach, we independently subjected the entire, unfiltered, set of expressed genes to 'Significance Analysis of Microarrays' (SAM) [69] in order to estimate a false discovery rate (FDR). When the FDR was set to 0.05, a set of 229 genes, which largely overlapped with the previous set of 246, was marked as translationally downregulated in *eif3h *and 73 genes were marked as upregulated.

### Quantitative real time PCR

Total RNA, polysomal and non-polysomal RNA were isolated as described above. We incubated 1 μg of DNase treated RNA and 0.5 μg of oligo(dT) primer at 70°C for 10 minutes and these were chilled on ice. To this mixture 4 μl of 5× reaction buffer, 40 units of RNasin (Promega), 1 μl of 20 mM each dNTPs, and 200 units of M-MLV reverse transcriptase (Promega) were added in a total volume of 20 μl. The reaction was incubated at 42°C for 50 minutes, and then inactivated at 70°C for 15 minutes. The first strand cDNAs were diluted to 100 μl, and 1 μl (1/100 of initial amount) was used for a 25 μl quantitative (Q)-RT-PCR reaction. Three replicates of Q-RT-PCR were performed using ABsolute™ QPCR SYBER Green Mix (ABgene, Surrey, UK) in a Bio-Rad iQ5 Real-Time PCR Detection System (Bio-Rad Laboratories, Hercules, CA, USA) according to the manufacturer's guide. Gene-specific oligonucleotide primers were: At3g08940, 5'-gtgtcatgtaatgatgtggtggc-3' and 5'-actgcgaggtataagaaattccg-3'; At2g46830, 5'-tctgatctgttgtttgtactctg-3' and 5'-tgaataatacagagtcaaatgttacagg-3'; At3g49910, 5'-gcagaacgttgattaagtaagaagg-3' and 5'-tagcaccataaagatccactgac-3'; At3g61650, 5'-aggaagaagcaagcttttctagac-3' and 5'-ccaacttcggatcaacaactcc-3'; At3g52590, 5'-accttgaccggcaagacca-3' and 5'-taagcctcaacacaagatgaagtg-3'; At4g05320, 5'-cacactccacttggtcttgcg-3' and 5'-ggtctttccggtgagagtcttc-3'; At5g66140, 5'-tctcaatcttgttttgactcgagc-3' and 5'-cttggtttgatcaagatcaagcg-3'; At5g54760, 5'-catggtttctaagctatctagtgg-3' and 5'-gaagaagtatggtgaacataagcca-3'; At4g34590, 5'-tttgtccgatttagagacatgtcc-3' and 5'-atgatgcttagagatacctaacgc-3'; At1g26670, 5'-gatggttgatttactttgtaatgg-3' and 5'-gacttgggaaaccttatgaatgt-3'; At1g29410, 5'-gtggtatttgcggcatagac-3' and 5'-agagtgcattattttgatgctcg-3'; At2g05710, 5'-aagcaatacacaagttatggagc-3' and 5'-aacccactgaaactcatatattgc-3'; At5g65430, 5'-ggtctaatggaagaaaagacgg-3' and 5'-caaagctgtgcacataatctgtc-3'. Reverse-transcriptase PCR analysis of FLUC and EF1α mRNA levels were performed as described [19,68].

### 5' UTR sequence analysis

Initial 5' UTR analyses in this paper were carried out using Microsoft EXCEL^® ^software. The 5' UTR sequences were downloaded from the TAIR website [70], which is based on the longest expressed sequence tag sequences. Among those sequences, only the sequences for genes listed in the SSP consortium's full-length cDNA list [34] were extracted to get the most reliable full-length sequences, and the resulting 12,129 genes were used for further analysis. The number of uAUGs as well as the length of each 5' leader sequence was determined. This dataset was used for analyzing our microarray result. The frequency distribution of the 5' leader length passed the Davis test for asymmetry; hence, the geometric mean was calculated. Expected triplet frequencies were calculated by generating a frame-independent dinucleotide frequency table from all 5' leaders. These dinucleotide frequencies were then used in a conditional probability formula to predict the expected triplet frequency, for example:

p(AUG) = p(AU) × p(UG)/(p(UG) + p(UA) + p(UT) + p(UC))

Real frequencies were then found in the manner of the previous dinucleotides, and the ratio of real to predicted was ascertained. Assorted uORF and leader properties were mined using the appropriate pattern matching scripts. All scripts were written in Perl and are available upon request.

## Additional data files

The following additional data are available with the online version of this paper. Additional data file 1 shows scatter plots of the microarray data. Additional data file 2 lists the genes identified as translationally or transcriptionally dependent on eIF3h. Additional data file 3 confirms the microarray-based polysome loading results by quantitative RT-PCR. Additional data file 4 lists the number of uAUGs for all *Arabidopsis *genes (AGI numbers) that are currently supported by full-length cDNA sequence. Raw microarray data were deposited at the Gene Expression Omnibus (NCBI GEO) under accession numbers < GSE6024 > for 'eif3h/WT polysome loading' and < GSE6025 > for 'eif3h/WT transcript level'. These 12 cel files are also submitted as Additional data files 5, 6, 7, 8, 9, 10, 11, 12, 13, 14, 15, 16. Additional data files 5, 6, 7, 8, 9, 10 correspond to the first experimental repeat and files numbered 11, 12, 13, 14, 15, 16 are the second experimental repeat. Files 5 and 12 are total RNA for *eif3h*. Files 6 and 11 are total RNA for wild type. Files 7 and 16 are polysomal RNA from *eif3h*. Files 8 and 15 are nonpolysomal RNA from *eif3h*. Files 9 and 14 are polysomal RNA from wild type. Files 10 and 13 are nonpolysomal RNA from wild type.

## Supplementary Material

Additional data file 1 Scatter plots of the microarray data. Click here for file

Additional data file 2 Genes identified as translationally or transcriptionally dependent on eIF3h. Click here for file

Additional data file 3 Confirmation of the microarray-based polysome loading results by quantitative RT-PCR. Click here for file

Additional data file 4 Number of uAUGs for all *Arabidopsis *genes (AGI numbers) that are currently supported by full-length cDNA sequence. Click here for file

Additional data file 5 Raw microarray data for total RNA for *eif3h *(first experimental repeat). Click here for file

Additional data file 6 Raw microarray data for total RNA for wild type (first experimental repeat). Click here for file

Additional data file 7 Raw microarray data for polysomal RNA from *eif3h *(first experimental repeat). Click here for file

Additional data file 8 Raw microarray data for nonpolysomal RNA from *eif3h *(first experimental repeat). Click here for file

Additional data file 9 Raw microarray data for polysomal RNA from wild type (first experimental repeat). Click here for file

Additional data file 10 Raw microarray data for nonpolysomal RNA from wild type (first experimental repeat). Click here for file

Additional data file 11 Raw microarray data for total RNA for wild type (second experimental repeat). Click here for file

Additional data file 12 Raw microarray data for total RNA for *eif3h *(second experimental repeat). Click here for file

Additional data file 13 Raw microarray data for nonpolysomal RNA from wild type (second experimental repeat). Click here for file

Additional data file 14 Raw microarray data for polysomal RNA from wild type (second experimental repeat). Click here for file

Additional data file 15 Raw microarray data for nonpolysomal RNA from *eif3h *(second experimental repeat). Click here for file

Additional data file 16 Raw microarray data for polysomal RNA from *eif3h *(second experimental repeat). Click here for file

## Figures and Tables

**Table 1:** Frequency of uORFs among *Arabidopsis *Gene Ontology classes

	Number of genes with uAUG					% uAUG-containing genes			
				
MapMan functional categories	0 uAUG	1 uAUG	2 uAUG	3 or more uAUG	Total genes	1 uAUG	2 uAUG	3 or more uAUG	% uAUG containing genes
Protein modification	183	79	52	71	385	20.52	13.51	18.44	52.47
Regulation	306	99	43	74	522	18.97	8.24	14.18	41.38
Cell division	22	7	3	5	37	18.92	8.11	13.51	40.54
Regulation of TC	804	244	126	155	1,329	18.36	9.48	11.66	39.50
Transport	312	97	46	39	494	19.64	9.31	7.89	36.84
DNA repair	13	3	2	2	20	15.00	10.00	10.00	35.00
Vesicle transport	38	11	0	9	58	18.97	0.00	15.52	34.48
Unknown	1,749	417	193	260	2,619	15.92	7.37	9.93	33.22
Cell organization	118	29	7	21	175	16.57	4.00	12.00	32.57
Development	201	44	23	30	298	14.77	7.72	10.07	32.55
Stress (abiotic)	177	43	19	14	253	17.00	7.51	5.53	30.04
Protein degradation	398	80	32	47	557	14.36	5.75	8.44	28.55
No ontology	1,298	276	114	126	1,814	15.21	6.28	6.95	28.45
Hormones	236	36	27	29	328	10.98	8.23	8.84	28.05
Stress (biotic)	101	17	9	12	139	12.23	6.47	8.63	27.34
Enzyme families	520	99	47	48	714	13.87	6.58	6.72	27.17
Protein targeting	87	15	6	4	112	13.39	5.36	3.57	22.32
DNA synthesis	103	16	6	6	131	12.21	4.58	4.58	21.37
RNA processing	114	21	5	3	143	14.69	3.50	2.10	20.28
AA activation	41	7	1	1	50	14.00	2.00	2.00	18.00
RNA synthesis	34	3	3	1	41	7.32	7.32	2.44	17.07
Cell cycle	44	5	3	1	53	9.43	5.66	1.89	16.98
Redox	121	15	3	6	145	10.34	2.07	4.14	16.55
Metal handling	57	4	2	3	66	6.06	3.03	4.55	13.64
Protein synthesis	340	31	6	7	384	8.07	1.56	1.82	11.46
