# Supplementary material for: On the functions of the h subunit of eukaryotic initiation factor 3 in late stages of translation initiation
Source: Genome Biol. 2007 Apr 17;8(4):R60. doi: 10.1186/gb-2007-8-4-r60 (PMC1896003; doi:10.1186/gb-2007-8-4-r60)
Supplement: Additional data file 1 — Scatter plots of the microarray data. [file gb-2007-8-4-r60-S1.pdf]

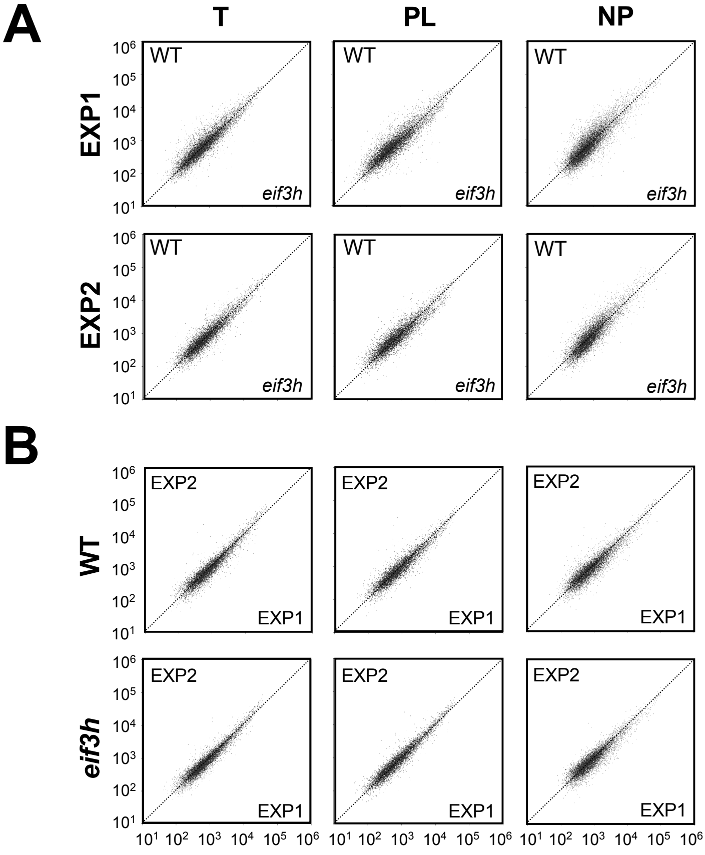

Figure S1. Scatter plots generated from microarray signal intensities after standard normalization. Genes with “absent” calls were excluded. (**A**) Comparison of signal intensities between wild type and *eif3h* mutant for total transcript (TC), polysome (PL) and non-polysome (NP) samples. **(B)** Comparison of signal intensities between two replicate experiments for total transcript levels (TC), polysomal (PL) and non-polysomal (NP) RNAs. The variance between experimental repeats (B) was smaller than that between wild-type and mutant samples (A), implying that the reproducibility between the two biological replicates was generally high.
