# Supplementary material for: On the functions of the h subunit of eukaryotic initiation factor 3 in late stages of translation initiation
Source: Genome Biol. 2007 Apr 17;8(4):R60. doi: 10.1186/gb-2007-8-4-r60 (PMC1896003; doi:10.1186/gb-2007-8-4-r60)
Supplement: Additional data file 3 — Confirmation of the microarray-based polysome loading results by quantitative RT-PCR. [file gb-2007-8-4-r60-S3.pdf]

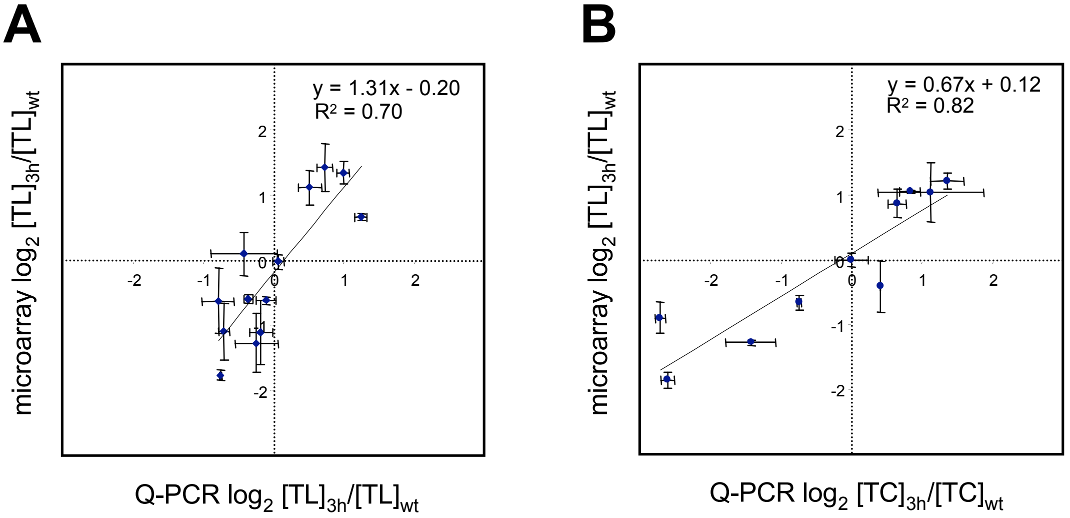

Figure S2. Quantitative reverse-transcriptase PCR analysis of polysome loading for representative mRNAs detected in the microarray experiment. Microarray data (y-axis) on polysome loading state [TL] or total mRNA levels [TC] are correlated with corresponding quantitative RT-PCR data (x-axis).
